# The role of the glycosyl moiety of myricetin derivatives in anti-HIV-1 activity in vitro

**DOI:** 10.1186/s12981-017-0183-6

**Published:** 2017-10-12

**Authors:** Joseph T. Ortega, Alirica I. Suárez, Maria L. Serrano, Jani Baptista, Flor H. Pujol, Hector R. Rangel

**Affiliations:** 1 Laboratorio de Virología Molecular, Centro de Microbiología y Biología Celular, Instituto Venezolano de Investigaciones Científicas, Caracas, Venezuela; 2 Laboratorio de Productos Naturales, Facultad de Farmacia, Universidad Central de Venezuela, Caracas, Venezuela; 3 Unidad de Química Medicinal, Facultad de Farmacia, Universidad Central de Venezuela, Caracas, Venezuela

**Keywords:** HIV-1, Reverse transcriptase, Flavonoids, Myricetin, Glycosyl flavonoids, HIV RT docking

## Abstract

**Background:**

Plant extracts are sources of valuable compounds with biological activity, especially for the anti-proliferative activity against pathogens or tumor cells. Myricetin is a flavonoid found in several plants that has been described as an inhibitor of Human immunodeficiency virus type 1 (HIV-1) through its action against the HIV reverse transcriptase, but myricetin derivatives have not been fully studied. The aim of this study was to evaluate the anti-HIV-1 activity of glycosylated metabolites obtained from *Marcetia taxifolia* and derived from myricetin: myricetin rhamnoside and myricetin 3-(6-rhamnosylgalactoside).

**Methods:**

Compounds were obtained from organic extracts by maceration of aerial parts of *M. taxifolia*. All biological assays were performed in the MT4 cell line. Antiviral activity was measured as inhibition of p24 and reverse transcriptase with a fluorescent assay.

**Results:**

Both flavonoids have antiviral activity in vitro, with an EC50 of 120 µM for myricetin 3-rhamnoside (MR) and 45 µM for myricetin 3-(6-rhamnosylgalactoside) (MRG), both significantly lower than the EC50 of myricetin (230 µM). Although both compounds inhibited the reverse transcriptase activity, with an IC50 of 10.6 µM for MR and 13.8 µM for MRG, myricetin was the most potent, with an IC50 of 7.6 µM, and an inhibition greater than 80%. Molecular docking approach showed correlation between the free energy of binding with the assays of enzyme inhibition.

**Conclusions:**

The results suggest that glycosylated moiety might enhance the anti-HIV-1 activity of myricetin, probably by favoring the internalization of the flavonoid into the cell. The inhibition of the HIV-1 reverse transcriptase is likely responsible for the antiviral activity.

## Background

The AIDS epidemic is the result of one of the most important viral infections affecting humans. As there is no vaccine currently available, the antiviral drugs constitute the only way to slow the progression of the disease [1, 2]. The appearance of drug resistance, serious side effects of existing drugs have promoted the search for new anti-HIV agents [3].

Plants are a rich source of new bioactive compounds [4]. Flavonoids, a large group of polyphenolic compound, are known for their bioactive properties and are widely distributed in the vegetal kingdom [5]. The range of their biological properties include anti-allergic, antibacterial, antidiabetic, anti-inflammatory and antiviral activities [6, 7].

The *Melastomataceae* are plants common in the tropical and mountainous areas. This family is abundantly distributed in South America, Southeast Asia and southern China [8]. *Marcetia* is a neotropical genus with 44 described species, distributed from Venezuela to Uruguay, with most of the species found in Southern Brazil and Uruguay [9]. The extract of *Marcetia taxifolia* has been shown to have antimicrobial activities, and the compounds responsible for this effect are flavonoids, particularly myricetin [10]. Pasetto et al. [11] and Ono et al. [12] determined that myricetin and quercetin inhibited HIV-1 proliferation and the HIV-1 reverse transcriptase (RT). The RT plays an essential role in the HIV life cycle and is one of the principal targets for several anti HIV-1 drugs in clinical use [13]. Some flavonoids have been characterized as nonnucleoside inhibitors (NNRTIs) that bind to a site in the p66 subunit of the HIV-1 RT p66/p51 heterodimer, situated approximately 10 Å from the RT active site [14, 15]. Ortega et al. [16], found that quercetin, a glycosylated derivative of myricetin, exhibited an improved antiviral activity and suggested that the enhanced activity was due to the glycosyl moiety.

**Fig. 1:**
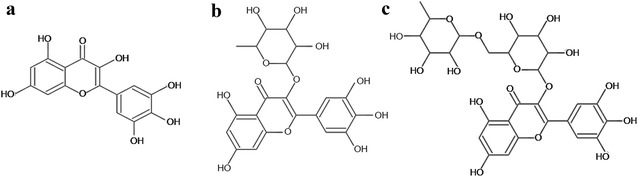
Chemical structure of the myricetin and derivatives **a** Myricetin (M), **b** myricetin 3-rhamnoside (MR) and **c** myricetin 3-(6-rhamnosylgalactoside) (MRG)

The aim of this study was to evaluate the anti-HIV-1 activity in vitro of glycosylated metabolites of myricetin obtained from *Marcetia taxifolia* extracts: myricetin 3-rhamnoside (MR) and myricetin 3-(6-rhamnosylgalactoside) (MRG). These compounds contain one and two glycosides respectively, as substituents on the myricetin backbone (Fig. 1). In order to determine the possible role of the glycosyl moiety on antiviral activity we used a molecular docking approach to compare the binding of the glycosylated compounds and the aglycone myricetin to HIV-1 RT.

## Methods

### Plant material and extraction

The plant *Marcetia taxifolia* (A.St.-Hil.) DC., was collected in the Amazonas State of Venezuela, and its botanical identity was confirmed and authenticated by Dr. Stephen Tillett. A voucher of the collection was archived with the code MYF 28418 in the “Herbario Víctor Manuel Ovalles” of the School of Pharmacy, Universidad Central de Venezuela. Extraction and separation of compounds obtained from the aerial parts of *M. taxifolia* was performed as described previously [17]. Two glycosylated compounds derived from myricetin: myricetin 3-rhamnoside and myricetin 3-(6-rhamnosylgalactoside) were evaluated, along with the commercially available aglycone myricetin (Sigma-Aldrich, USA).

### Cells and virus

HIV-1 (HTLV-IIIB/H9) and MT4 cells were obtained from the NIH AIDS Research and Reference Reagent Program. The cells were grown in RPMI-1640 medium supplemented with 10% FBS and penicillin/streptomycin and splitted every 3 days.

### Cytotoxicity assay

MT4 cells were seeded in 96 wells/plate at a density of 30,000 cells/well, and different concentrations of the compounds were added. After 24 h the cultures were evaluated with MTT cell proliferation assay (Sigma-Aldrich, USA) to determine the percent of live cells under the different conditions evaluated.

### Antiviral activity

Different concentrations of the compounds and HIV-1, at an MOI of 0.03, were added simultaneously to the wells of a 96 wells plate containing 30,000 cells/well. The virus production was evaluated at 72 h post infection by detecting the viral antigen p24 with an in-house ELISA [18]. The results were expressed relative to the control of untreated HIV-1 infected cells. Selectivity Index value (SI) was determined as the ratio of CC50 to EC50 for each compound.

### RT inhibition assay

The inhibitory activity of compounds against HIV-1 RT was evaluated by using the EnzChek Reverse Transcriptase Assay kit (Molecular Probes, Inc., USA). Purified RT enzyme purchased from Worthington Biochemical Corporation and Nevirapine were used as controls for enzyme and antiviral drug respectively. Statistical analysis (ANOVA) of the results from at least three independent experiments was performed using Prism GraphPad 6 version 6.01 (p ≤ 0.05 was used as the level of significance).

### Molecular docking

The coordinates of wild-type HIV-1 reverse transcriptase (RT) bound with DNA and the NNRTI Nevirapine at 2.85 Å resolution was obtained from the protein data bank [19], PDB code 3V81 [20]. The inhibitor, the DNA molecule and all crystallographic water molecules were removed from the coordinate set: hydrogen atoms were added and partial charges were assigned to all atoms. The enzyme was then submitted to restrained molecular mechanics refinement with NAMD [21], using the CHARMM22 force field. The binding site was delimited according to previous studies reporting key residues of the enzyme and the 3D structure of each inhibitor was obtained from the PubChem database [22]. The molecular docking was performed with AutoDock 4/VegaZZ 3.1.0.21 and 30 runs were conducted for each compound. The results were prioritized according to the predicted free energy of binding in kcal/mol.

## Results

### Cytotoxicity and antiviral activity

**Fig. 2:**
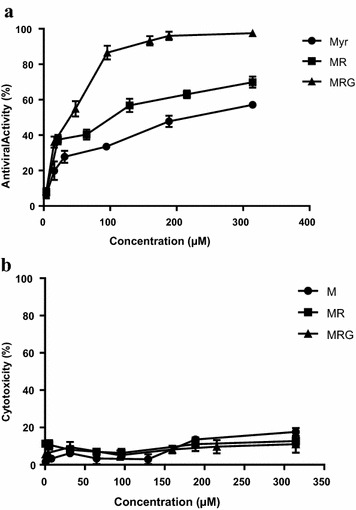
Antiviral **a** and cytotoxic **b** activity of compounds evaluated. The percent of cytotoxicity of different concentrations of MR (Square), MRG (Triangle) and M (Circle) are shown as media ± SEM. Antiviral activity (solid lines) is expressed as the percent inhibition of p24 production relative to control. Results are media ± SEM (n = 3)

The anti-HIV-1 activity in vitro was evaluated with a wide range of concentrations of the myricetin glycosylated compounds. The EC50 for myricetin 3-rhamnoside was 120 µM, for myricetin 3-(6-rhamnosylgalactoside) 45 and 230 µM for myricetin (Fig. 2a). Neither myricetin nor either of the myricetin derivatives used, exhibited significant cytotoxic activity at the concentrations tested (Fig. 2b). Because the CC50 for all the compounds assayed was out of the range of the evaluated concentrations, (maximum > 300 µM) an exact estimation of the selectivity index (SI) was impossible, and instead a crude estimation was performed by assigning the CC50 as > 300 µM and then calculating the EC50 for each compound. The estimated CC50 values thereby obtained were: IS_M_ > 1.3¸ IS_MR_ > 2.6 and IS_MRG_ > 7.

### RT inhibition assay

**Fig. 3:**
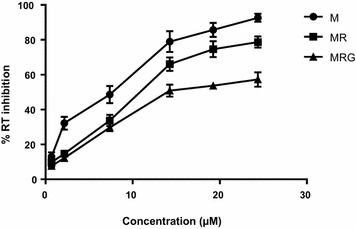
Reverse transcriptase inhibition. The inhibitory effect of MR (Square) MRG (Triangle) and M (Circle) on recombinant Reverse Transcriptase enzyme (0.25 UI/ml), is expressed as the activity of the treated enzyme relative to control w/o treatment. All results were normalized to respective control (reaction buffer)

The capacity of the compounds to inhibit HIV-1 RT was also evaluated. Figure 3 shows that at a concentration higher than 7.5 µM, myricetin 3-rhamnoside was more potent than myricetin 3-(6-rhamnosylgalactoside), reaching an inhibitory effect over 65%. However, the aglycone myricetin exhibited more RT inhibition at all concentrations evaluated. The IC50 obtained for myricetin was 7.6 µM, while for myricetin 3-rhamnoside it was 10.6 µM, and for myricetin 3-(6-rhamnosylgalactoside) 13.8 µM.

### Molecular docking

**Fig. 4:**
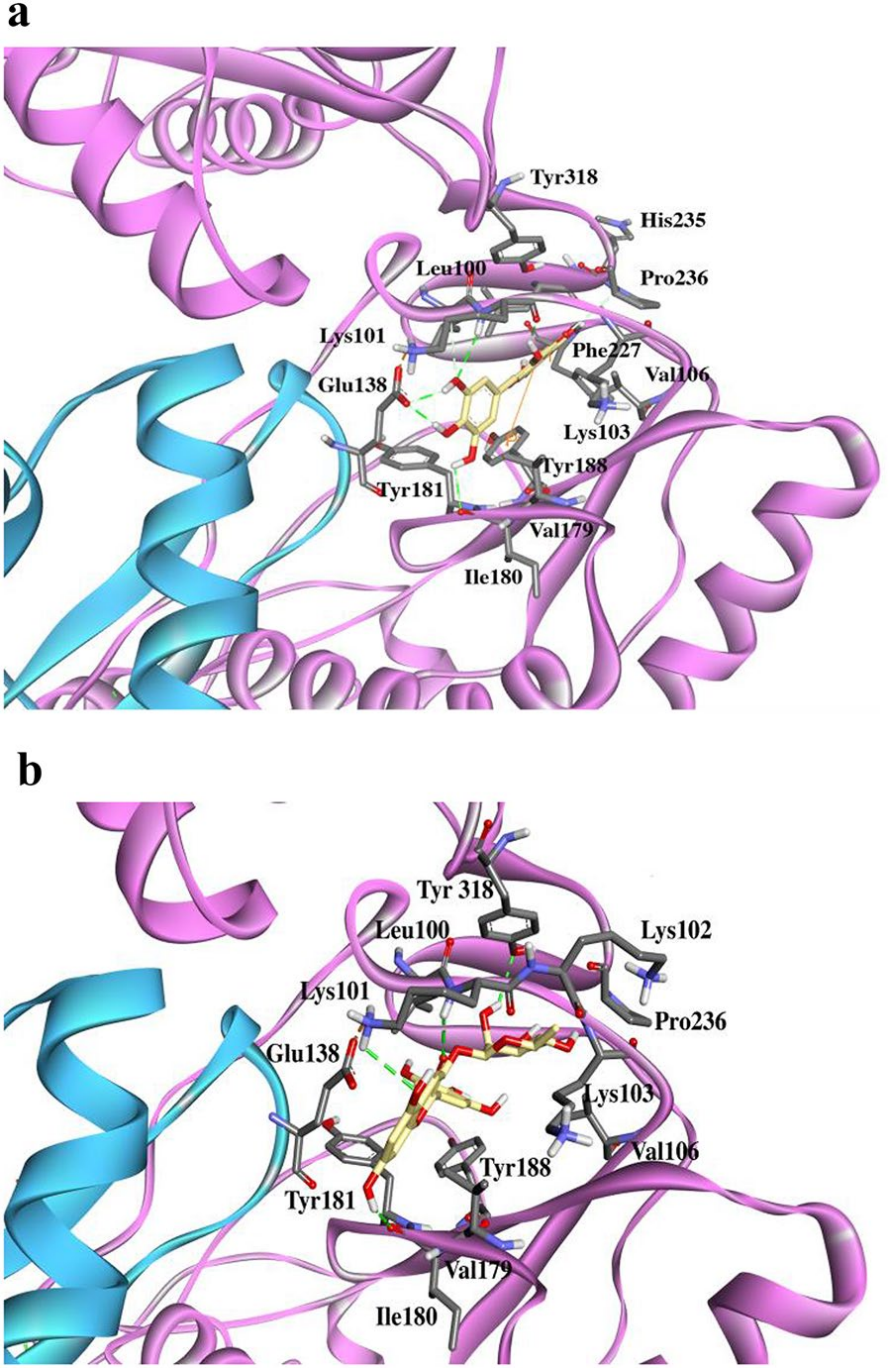
Docking results. Best pose of M **a** and MR **b** into the no nucleoside binding pocket of HIV-1 RT. The ligand is shown in gold and residues involved in the interactions in gray. The hydrogen bonds are represented as a green dotted line

The potential interactions of the active compounds with the RT enzyme were evaluated by molecular docking. Myricetin exhibited the lowest free energy of binding (− 6.95 kcal/mol), compared to glycosylated compounds (Table 1). In Fig. 4, which shows the interactions between myricetin and RT residues, there are five hydrogen bonds with residues Lys101 (A), Glu138 (B) and Ile180 (A) and electrostatic interactions Pi–Pi, Pi–Alkyl and Pi–sigma with residues Tyr188 (A), Leu100 (A), Val106 (A), Leu234 (A) and Val179 (A). Of the two myricetin glycosides, myricetin 3-rhamnoside exhibited the best free energy of binding to RT, (− 5.02 kcal/mol) and Fig. 4b shows the interactions of myricetin 3-rhamnoside with various amino acid residues in the binding pocket of RT NNRTI inhibitors. Four hydrogen bonds with residues Tyr318 (A), Lys101 (A) and Ile108 (A) were observed, along with electrostatic interactions such as Pi–Pi, Pi–Alkyl, Pi–sigma and Pi–anion with Tyr181 (A), Val 179 (A), Leu100 (A), Glu 138 (B), and Alkyl–Alkyl interactions with the residues Lys103 (A), Val106 (A), Val179 (A) and Pro236 (A). Myricetin 3-(6-rhamnosylgalactoside) showed a higher free energy of binding to RT (2.38 kcal/mol) and fewer interactions than seen with myricetin 3-rhamnoside, two hydrogen bonds with residues Lys101 (A) and Glu138 (A) and several electrostatic interactions Pi–cation and, Pi–Alkyl that occurred with the residues Leu100, Pro321, and Lys101.

**Table 1:** Docking analysis of the evaluated compounds

Mol ID	EC_50_ (Elisa p24)	EB^a^	RT interactions	Residues (H-bonds) Number of H bonds
M	230	− 6.95	vdW: Lys102 (A), Lys103 (A), Tyr181 (A), Phe227 (A), His235 (A)	Lys101 (A), Ile180 (A) Glu138 (B), Leu100 (A), Pro236 (A)
			Pi interactions: Leu100 (A), VAL106 (A), Tyr188 (A), Val179 (A), Leu 234 (A)	*5*
MR	120	− 5.02	vdW: Pro95 (A), Lys102 (A), Thr139 (B), Lys172 (A), Tyr188 (A), Phe227 (A), Trp229 (A), Leu234 (A), His235 (A)	Lys101 (A), Ile108 (A), Tyr318 (A)
			Pi interactions: Leu100 (A), Tyr181 (A), GLU138 (B), VAL179 (A)	*4*
			Alkil interactions: Lys102 (A), Val106 (A), Pro236 (A)	
MRG	45	2.38	vdW: Glu 28 (B) Lys 102 (A), Lys 103 (A), Val 106 (A), Ile 135 (A), Val 179 (A), Tyr 181 (A), Tyr 188 (A), Gly 190 (A) Asp 192 (A), Asp 320 (A)	Lys101, Glu138
			Pi interactions: Leu 100 (A), Pro 321 (A)	*2*

*M* myricetin, *MR* myricetin rhamnoside, *MRG* myricetin 3-(6-rhamnosylgalactoside). EC50: Antiviral activity (µM) measured by Elisa p24, *EB* free energy of binding (kcal/mol), *vdW* van der Waals interactions

^a^Note that the more positive the free energy of binding, the less likely the probability interaction

## Discussion

Because of several reports describing the activity of other flavonoids against HIV-1 [23–25] we evaluated the activity of glycosylate flavonoids obtained from *M. taxifolia*. The results showed that the antiviral potency of myricetin derived compounds was related to the number of glycosyl residues on the molecule supporting the notion that the addition of glycosides to myricetin could modulate the anti-HIV-1 activity of these compounds.

Flavonoids are generally found in plants and fruits as glycosides and less commonly as aglycones [26]. The glycosilated flavonoids obtained from the diet are hydrolyzed in the intestine and then transported into the cells and systemic circulation. However, it has been described that glycosylated flavonoids may be a substrate of sodium-dependent glucose transporters (SGLT1) in the cell membrane of enterocytes [27, 28] and then, once inside the cell, the glycosides are cleaved by a cytosolic beta-glycosidase [29]. This process could contribute to their accumulation within the cell. Consistent with this possibility, it has been shown that two glycosyl derivatives of quercetin (guajaverin and avicularin) have higher antiviral activity than quercetin [16]. Similar results were obtained in this study, with a reduction of two to fivefold of the EC50 with the addition of glycosyl moieties to myricetin.

In contrast to the results observed with the inhibition of HIV-1 replication, RT inhibition was inversely proportional to the glycosylation grade of myricetin [myricetin (R = aglycone) > myricetin 3-rhamnoside (R = mono-glycosylated) > myricetin 3-(6-rhamnosylgalactoside) (R = Bi-glycosylated)]. These results are in concordance with the free energy of binding obtained in silico, where myricetin exhibited the lowest free energy and MRG the highest. The analysis of the resulting complexes clearly reveals that the binding interactions of these compounds occurred with the residues of the hydrophobic NNRTI-binding pocket, described by Sarafianos et al. [30] as containing residues Leu100, Lys101, Lys103, Val106, Tre107, Val108, Val179, Tyr181, Tyr188, Val189, Gly190, Phe227, Trp229, Leu234, and Tyr318 of the p66 sub-unit and Glu138 of the p51 sub-unit. The analysis of the docked complexes emphasizes the relevant role of the H-bonds formed with residues Lys101, Ile 108, Ile 180, Tyr 318 and Glu 318. The results presented in this work are in agreement with those obtained by Syahdi in 2012 [31], which evaluated HIV RT docking of other flavonoids derived from plants and found that a hydrogen bond interaction with Lys101 is present on the docked pose with the lowest free energy of binding. From the docking analysis, myricetin might interact through five hydrogen bonds, while myricetin 3-rhamnoside through four hydrogen bonds and myricetin 3-(6-rhamnosylgalactoside) through only two. The number of hydrogen bonds formed is related to the free energy of binding found for each compound, and those molecules that can form a greater number of hydrogen bonds consequently create a more stable interaction.

Myricetin and the derivatives evaluates in this study have not been tested against HIV-1 resistant strains, nor mutant enzymes, in vitro to evaluate their capacity to inhibit viral replication of NNRTI resistant viruses. However, the docking study of myricetin on a RT enzyme with mutations conferring NNRTI resistance (L101I, K103N, and E138K) was performed with the protein models 1s1u, 1fkp and 2hny respectively. The analysis showed that the mutations did not result in significant changes in the free energy of binding levels, although with the L101I mutation, a small change in the orientation of myricetin in the binding pocket was observed. To better understand the possible effect of mutations, our laboratory is currently studying the interaction of myricetin in the NNRTI binding pocket through molecular dynamics simulations. We will also perform an in vitro analysis with NNRTI resistant strains of HIV-1 to determine the effect of the mutations on inhibitory action of these flavonoids derivatives.

## Conclusions

The flavonoids are produced naturally as glycosides in plants and fruits but are found as aglycones in blood, as a consequence of the hydrolysis by glycosidases at the intestine lumen or inside the cell. The glycosylated flavonoids could be actively transported by SGLT1, with posterior hydrolysis within cytoplasm or they could be hydrolyzed at the cell surface and then enter the cell by diffusion. The existence of two different chemical forms of the flavonoid could generate a gradient constant favoring an increased concentration inside the cell.

The differences in the antiviral activity and the RT inhibition of the myricetin, MR and MRG together with the in silico analysis suggest that the glycosyl moiety could play a role in the entry of flavonoids into the cell and then, after enzymatic cleavage of the glycosyl moiety, the myricetin aglycone would ultimately be responsible for anti-HIV activity. This activity is presumed to be mediated primarily by the inhibition of RT, although interaction with other targets can’t be discarded with the results described. Further studies are required to clarify the role of the glycosides in the activity/transport of myricetin and other related flavonoids.

## Abbreviations

HIV: human immunodeficiency virus
RT: reverse transcriptase
MOI: multiplicity of infection
M: myricetin
MR: myricetin 3-rhamnoside
MRG: myricetin 3-(6-rhamnosylgalactoside)
EC50: half effective concentration
CC50: half cytotoxic concentration
IC50: half inhibitory concentration
